# Treatment of mild and moderate type-2 diabetes: open prospective trial with *Hintonia latiflora* extract

**DOI:** 10.1186/2047-783X-19-16

**Published:** 2014-03-28

**Authors:** Marta Korecova, Marie Hladikova

**Affiliations:** 1 Head of Diabetes Department, IDF President, Rc: 425201/734, Vel’komoravská 2, Trencin, SK 91101, Slovak Republic; 2 Department for Medical Informatics, 2nd Medical Faculty of Charles University of Prague, V Úvalu 84, Prague 5 CZ-15006, Czech Republic

**Keywords:** *Hintonia latiflora*, Type 2 diabetes, HbA_1c_, Blood glucose, Liver values, Blood lipids

## Abstract

**Background:**

Extracts from the bark of *Hintonia latiflora* are used as dietetic measures to support the regulation of glucose metabolism and the stabilization of blood glucose values.

**Methods:**

A dry concentrated extract from the bark of *Hintonia latiflora* in capsule form was tested in an open, prospective clinical study in 41 dietetically stabilized subjects with type 2 diabetes. The effects on parameters of blood glucose control were documented over a period of six months.

**Results:**

Fasting and postprandial glucose and the HbA_1c_ value declined significantly. In the case of HbA_1c_, this meant a reduction of the absolute value from 7.49 ± 0.72% to 6.82 ± 0.67% (from 58.4 to 51.0 mmol/mol Hb; intention to treat (ITT) population). Furthermore, cholesterol and triglycerides were slightly reduced and no negative effect on other laboratory parameters and no change of the liver values were observed. Tolerance was very good. In particular, no side effects and no hypoglycemic episodes or worsening of diabetic symptoms occurred.

**Conclusions:**

The study confirms the positive effect of extracts from the bark of *Hintonia latiflora* on blood glucose values suggesting a potential benefit in the management of glucose metabolism in cases of type 2 diabetes.

**Trial registration:**

Reg.-No. ISRCTN83308122

## Background

Positive effects of preparations of the bark of the Central American plant *Hintonia latiflora* (family Rubiaceae) on blood glucose reduction and therefore the maintenance of physiologically normal blood glucose values have been reported in scientific investigations published over at least the past 60 years [1-8]. Normalizations of diabetic parameters, with the absence of hypoglycemic episodes, have been observed upon exposure to *Hintonia* extracts [9-13]. In a long-term study lasting up to 33 months, the mean HbA_1c_ value of the participants decreased below the target value of 7% after six months of consumption of a liquid extract from the bark of *Hintonia latiflora*, with excellent tolerance [14].

The basic hypothesis of the present six-month open, prospective study was that the previously observed antidiabetic effects can be achieved with an alternative preparation of the *Hintonia* extract in the form of capsules from a dry concentrate. The selected dose scheme corresponded to the patient exposure in the previous study. It was known to be safe from more than 30 years of clinical experience with the liquid formulation, and from the mentioned bibliographic sources. Safety in the relevant dose range has also been established through animal studies with chronic exposure [4].

## Methods

### Study design and test preparation

The study was designed as a six-month, prospective open prospective clinical study. It was based on the ethical principles of the Declaration of Helsinki/Somerset West and registered with ISRCTN (register number ISRCTN83308122). Signed informed consent was required from the study participants.

The test preparation of this study (Sucontral™ D Capsules, Harras Pharma Curarina, Munich, Germany) contained 100 mg of a dry concentrate per capsule (extraction solvent ethanol 32%; DER_final_ 2.4:1) from the bark of the Central American plant *Hintonia latiflora* (Sessé & Moc. ex DC.) Bullock (Rubiaceae). The plant material was from a controlled collection. The extract was prepared under good manufacturing practice (GMP) conditions and tested against a predefined analytical specification including tests for heavy metals, aflatoxins, pesticides and microbiology as well as an assay on polyphenol content (6 to 10%, calculated as coutareagenin). Per capsule, 24 mg polyphenols were supplied. The capsules also contained 30 mg vitamin C, 5 mg vitamin E, 0.7 mg vitamin B_1_, 0.8 mg vitamin B_2_, 1 mg vitamin B_6_, 0.5 μg vitamin B_12_, 100 μg folic acid, 75 μg biotin, 2.5 mg zinc and 25 μg chromium. In accordance with the recommended intake, patients took one capsule before meals twice daily while adhering to a prescribed diet.

Both the stabilization of the study participants to constant HbA_1c_ values, and the testing of diabetic parameters took place in the study center. Only subjects suffering from type 2 diabetes in the age range of 45 to 80 years, taking neither oral antidiabetic agents nor insulin, were eligible as participants. All participants followed a dietary regime, which led to stable, but not normoglycemic values.

For inclusion in the study, diabetic symptoms must have existed for a period of at least 12 months. The values for fasting blood glucose were to be in the range of 7 to 14 mmol/l (normal value: 3.9 to 5.4 mmol/l).

Doubts regarding the reliability of a potential participant with respect to the adherence to the dietetic regime (for example, with respect to alcohol consumption) were defined as an exclusion criterion. Other reasons for exclusion were severe diabetic symptoms, progressive life-threatening diseases, hepatic dysfunction or renal insufficiency (deviations of GOT, γGT and AP of more than twice the normal value, serum creatinine >130 μmol/l), hypoglycemic crises not noticed in time, retinopathy, pregnancy, malignant tumors and/or drug or alcohol dependence in the patient’s medical history.

The dietary measures continued unchanged over the entire course of the study to exclude a distorting influence of nutrition on the study results confirming the attribution of changes of the diabetic parameters to the tested preparation containing a dry concentrate from *Hintonia* bark.

Anamnestic and demographic data, diabetic parameters and liver values were collected at the start of the study. Fasting and postprandial blood glucose (two hours after food intake), blood pressure and body weight were recorded at monthly intervals. Indications of improvements or deteriorations of the diabetic symptoms (neuropathy, paraesthesia, constipation and sweating) were likewise documented at monthly intervals. The HbA_1c_ value, liver function tests and blood lipids were recorded at three and six months.

### Biometric analysis

Statistical differences were assessed by ANOVA tests for repeated measurements. The statistical software package was SPSS version 20, release 20.0.0.

The statistical significance values were calculated both on the ‘per protocol’ population (PP, all participants who completed the study as planned) and the ‘intention to treat’ population (ITT; with the last measured value carried over in cases of lacking data).

## Results

The demographic data of the patients is presented in Table 1. Forty-one participants were included in the study. Nine of the participants (two men and seven women) prematurely terminated their participation in the study:

**Table 1:** Demographic and anamnestic patient data

**Number of patients**	**n = 41 (100%)**
Female	n = 33 (80.5%)
Male	n = 8 (19.5%)
Age	60.9 ± 8.5 years
Age range	41 to 79 years
Duration of the diabetes symptoms	5.9 ± 4.9 years
Range for duration of the diabetes symptoms	1 to 19 years

1) at the participant’s request in four cases (two after one month, and two after three months); 2) on recommendation of the physician in four cases due to unreliability of the participant with respect to compliance with the dietary measures: (two after one month, and one after two and three months, respectively); and 3) in one case because of an acute infection from a concomitant disease.

All nine participants had shown improvements of the diabetic parameters at the time of withdrawal. This included HbA_1c_ values for all dropouts with a minimum study duration of three months.

The size of the ITT population was n = 41, and that of the PP population was n = 32 participants.

### Effect on diabetes parameters

The results of the statistical analysis differed only slightly for the ITT and PP population. For both analyses, there was a statistically significant difference compared with the starting values for all time points where measurements were made (*P* <0.001 in all individual cases).

**Figure 1:**
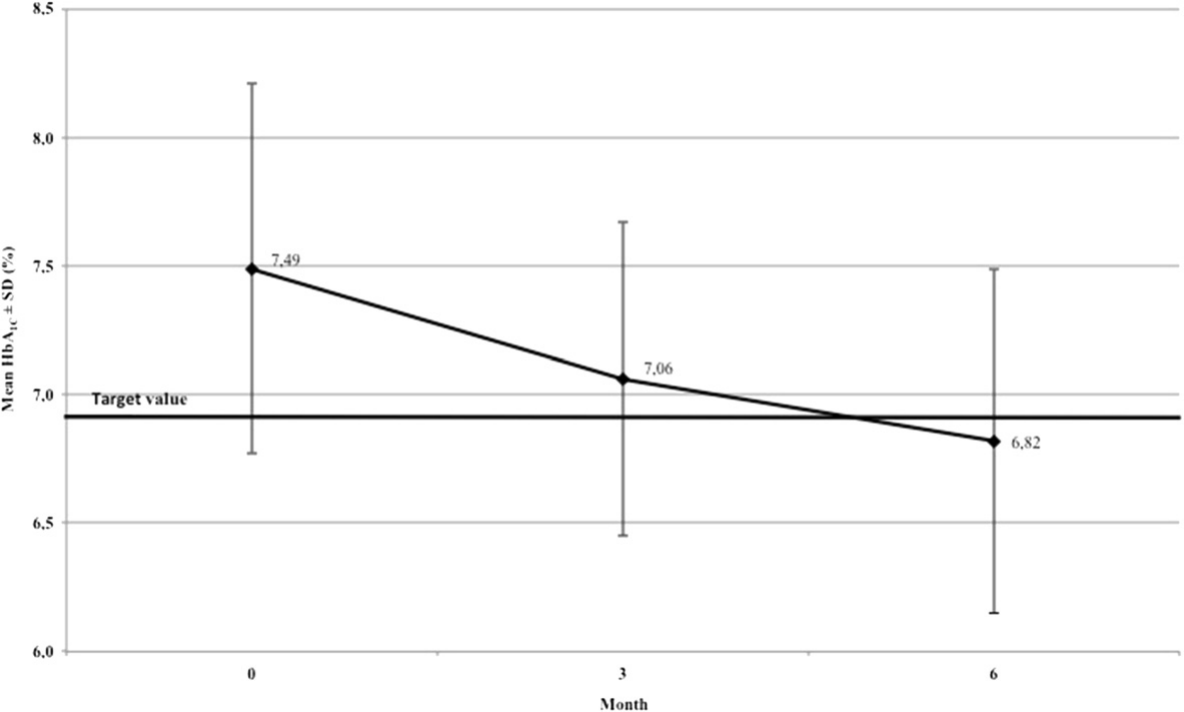
**Course of HbA**_**1c **_**values under intake of *Hintonia *extract.** Course of HbA_1c_ values in patients during treatment with *Hintonia* extract. All measured values were statistically significant compared with the starting value (*P* <0.001).

The average HbA_1c_ value at the start of the study was 7.49 ± 0.72% (58.36 mmol/mol Hb). Within the six months of exposure, the HbA_1c_ value had decreased by 8.95% (ITT population) and 11.2% (PP). In absolute values, this corresponded to a decrease by 0.67% (7.32 mmol/mol Hb) in the ITT group to a final value of 6.82 ± 0.67% (51.04 mmol/mol Hb), and of 0.84% (9.18 mmol/mol Hb) in the PP population to a final value of 6.65 ± 0.58% (49.18 mmol/mol Hb). The target value of 7% (53 mmol/mol Hb) as predefined upper limit for clinical relevance was therefore reached (Figure 1).

**Figure 2:**
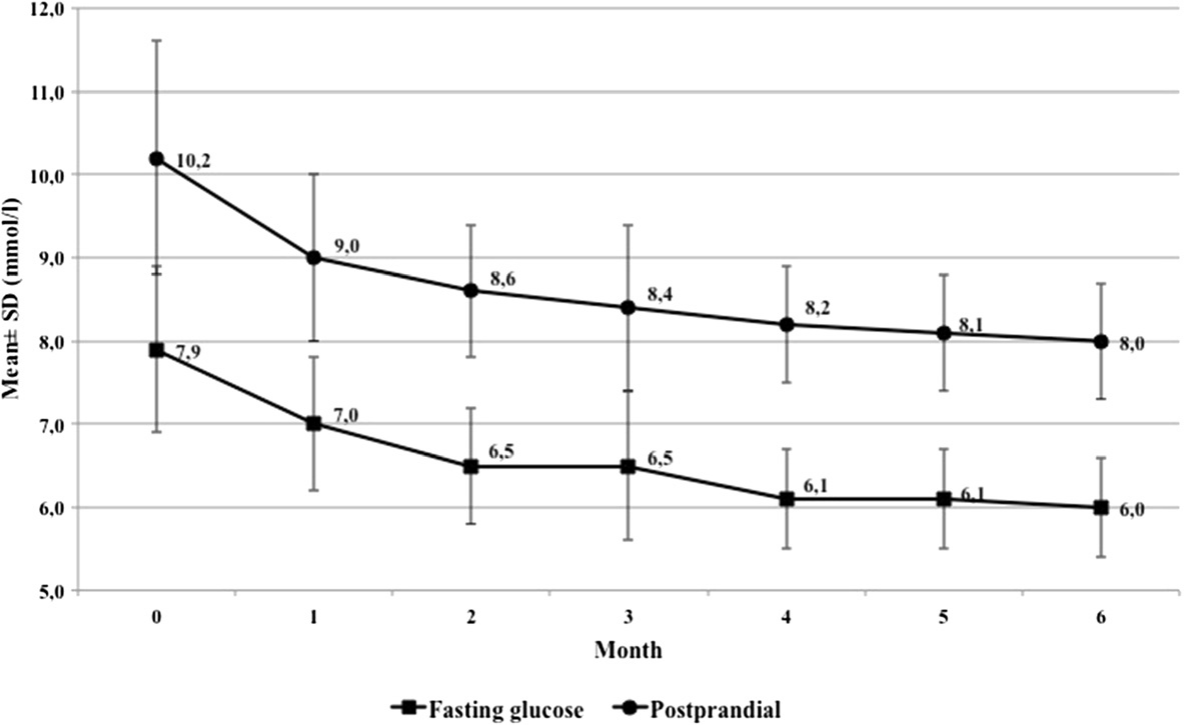
**Course of fasting glucose and postprandial blood glucose under intake of *Hintonia *extract.** Course of fasting glucose (squares) and postprandial blood glucose (circles) in patients during treatment with *Hintonia* extract. All measured values were statistically significant compared with the starting value (*P* <0.001).

Similar results were found for fasting and postprandial glucose; again, the six-month exposure resulted in a statistically significant reduction of blood glucose over starting values, with a continuous improvement of values at every monthly measurement. Both, the ITT and the PP analysis yielded practically identical results with a 25% reduction of starting values of fasting blood glucose (from 8.0 ± 1.0 to 6.0 ± 0.6 mmol/l in both groups), and a 22% lowering of the postprandial glucose (from 10.2 ± 1.4 to 8.0 ± 6.7 mmol/l) (Figure 2).

### Liver parameters and blood lipids

The laboratory parameters were only considered in the ITT analysis. All parameters remained uninfluenced, with the exception of a slight improvement of the values of the transaminases ALT and GGT. This improvement was found to be statistically significant, but not clinically relevant; the value of alanine aminotransferase ALT decreased from 0.41 ± 0.10 U/l to 0.36 ± 0.07 U/l, and that of glutamyl aminotransferase from 0.45 ± 0.29 U/l to 0.36 ± 0.17 U/l. However, the starting values were already clearly within the ‘healthy’ range (0 to 35 U/l). Other liver values and clinical laboratory tests such as alkaline phosphatase, aspartate aminotransferase, bilirubin or creatine kinase remained unchanged. For the blood lipids, which were found to be elevated at the start of the study, a slight but clinically marginally relevant decrease was noted; cholesterol decreased from 213.5 ± 34.0 mg/dl to 202.6 ± 32.9 mg/dl (p = 0.026), and triglycerides from 193.1 ± 87.7 mg/dl to 164.8 ± 59.3 mg/dl. Other parameters such as LDL and HDL showed no change.

## Discussion

The three crucial glycemic parameters, HbA_1c_, fasting and postprandial glucose, were statistically significantly and clinically reduced during intake of a dry concentrate from the bark of *Hintonia latiflora*. Published studies suggest a risk reduction of diabetic macro- and microangiopathies with an improvement of glycemic parameters [15,16].

The so-called ‘ADVANCE’ study observed a positive effect of an improved control of HbA_1_c on risk factors of diabetes mellitus through the application of oral antidiabetics [17]. In parallel, there was another large study commonly designated as the ‘ACCORD’ trial, where surprisingly, an aggressive lowering of HbA_1c_ by a combination of antidiabetic medications was associated with an increased long-term mortality [18]. It has been suggested that this seemingly paradoxical finding might have been caused by pre-existing cardiovascular risk factors in patients with insufficiently controlled long-term diabetes, by adverse effects of the medication, and, last but not least, by hypoglycemic episodes triggered by the study medication(s) [19,20]. Clearly the debate around the two studies shows that measures for an early improvement of glycemic control and homeostasis are warranted, especially if they are not associated with a risk of hypoglycemic episodes. Hintonia bark extract is allocated to the context of basic measures. According to its clinical characteristics known from case reports and clinical observations Hintonia extract could possibly postpone the time point for the requirement of therapy with oral antidiabetics, or even contribute to a reduction of the dose of the antidiabetic medication. Both may be considered a positive effect in the light of the ADVANCE and ACCORD studies, although the latter point has not been explicitly examined in this study.

The lack of a control group could be questioned with respect to the study design. In the case of diabetes mellitus, however, placebo effects do not play a role in the long-term application, and the measured laboratory values are objective parameters. Untreated type 2 diabetes is typically a progressive disease without a tendency to spontaneous improvements. In long-term studies with oral antidiabetics, the HbA_1c_ value remained unchanged in the placebo group, or even showed an increase [21-23]. In the ‘United Kingdom Prospective Diabetes Study’, the HbA_1c_ value remained stable under the diet only during the first year, and subsequently worsened continually. With severely overweight patients, the HbA_1c_ values increased from the start in spite of the diet [24]. A recently published long-term lifestyle intervention examined the effects of decreased caloric intake on the cardiovascular risk of obese patients with type 2 diabetes. Again, the laboratory results for glycated hemoglobin slowly increased over time in the control group despite a small loss in body weight, whereas the initial improvement in the intervention group was almost lost with a re-increase of body weight [25].

The results of these studies confirm that placebo effects under treatment with antidiabetic agents would not be expected, which justifies an open trial design for studies examining the potential of an intervention.

The participants of the present study were initially treated with dietary measures until no more improvement of HbA_1c_ was achieved. The starting values at the beginning of the study were the result of a dietary adjustment. This dietary regimen was consistently complied with. The dietary measures alone were insufficient to achieve approximately normoglycemic values, the values were, however, not sufficiently elevated as to justify an intervention with blood glucose lowering drugs such as insulin or oral antidiabetics. As the diet was continued unchanged during the entire study, and therefore did not represent a variable, the reduction of the diabetic parameters were not attributable to nutritional effects, but rather the result of the intake of the study preparation. The measurement of objective parameters without the explicit use of a control group therefore allows drawing valid assertions regarding the effects of the test preparation.

The test preparation was a dry concentrate from the bark of *Hintonia latiflora*, with a supplement of vitamins and trace elements that in turn can also play a role in the carbohydrate metabolism. Consequently, the question could be raised whether these additional substances may have contributed to the study result. A slight supplementary effect on blood glucose balance must in fact be expected from the administration of vitamins, and above all from trace elements such as chromium. However, the extent of the observed improvement is unlikely to be reached by vitamins and trace elements alone. A comparable effect was previously achieved with the long-term administration of extract from the bark of *Hintonia latiflora* in human beings without the addition of other micronutrients [14], which confirms the nutritional physiological importance of the plant preparation. Furthermore, positive effects on the regulation of the blood glucose balance could also be experimentally verified and attributed to the polyphenolic fraction [8].

## Conclusions

The intake of a neoflavonoid-containing dry concentrate from the bark of *Hintonia latiflora* improved the parameters of blood glucose control in type 2 diabetes mellitus. The tolerance was excellent, and liver and lipid values tended to be positively affected.

The observations of this study are consistent with earlier studies, and specifically with a controlled long-term study. The use of *Hintonia* dry extract in the dietetic treatment of disturbances of glucose balance in cases of mildly to moderately severe type 2 diabetes can accordingly be regarded as safe and useful in those cases, where an adequate control of the blood glucose balance cannot be achieved with dietary measures alone.

## Abbreviations

GMP: good manufacturing practice; ITT: intention to treat population; PP: per protocol population.

## Competing interests

M.K. and M.H. declare that they have no conflict of interests. The study was financially supported by Gehrlicher Extrakte, Germany. The sponsor also provided the test preparation. The authors alone were, however, responsible for the performance of the study, the evaluation and the publication of the results.

## Authors’ contributions

MK did the cli research. MH made the statistical evaluation and prepared the study report. MK is the guarantor of this work and, as such, had full access to all the data in this study and takes responsibility for the integrity of the data and the accuracy of the data analysis. Both authors read and approved the final manuscript.

## Authors’ information

M.K.’s clinical focus in on diabetes treatment and management, which includes the long-term practical implications of the exposure of patients to antidiabetics. M.H.’s field of expertise is the biometrical analysis of clinical study data.
